# Cutis marmorata telangiectatica congenita: a literature review

**DOI:** 10.1186/s13023-019-1229-8

**Published:** 2019-12-04

**Authors:** Teresa Nu Phuong Trinh Bui, Ayse Corap, Anette Bygum

**Affiliations:** 1 Department of Dermatology and Allergy Centre, J.B. Winsløws Vej 4, Entrance 142, 5000 Odense C, Denmark; 2 Department of Clinical Genetics, J.B.Winsløws Vej 4, Entrance 24, 5000 Odense C, Denmark

**Keywords:** Cutis marmorata telangiectatica congenita, CMTC, Van Lohuizen syndrome, Glaucoma, Leg length discrepancy, Associated anomalies

## Abstract

**Background:**

Cutis marmorata telangiectatica congenita (CMTC) is a rare capillary malformation characterised by persistent reticulated marbled erythema. It tends to be associated with cutaneous atrophy, ulcerations and body asymmetry. CMTC is usually reported to be a benign condition; however, associated anomalies are not rare. Here, we have compiled information on published CMTC patients with the aim to evaluate the proposed diagnostic criteria by Kienast et al. and address the clinical manifestations, associated anomalies, differential diagnoses, management and prognosis. Our review is based on a search of the PubMed database which retrieved studies between 1922 and April 2019. The search yielded 148 original articles with a total of 485 patients.

**Results:**

Of the identified patients, 24.5% had generalised CMTC, 66.8% had localised and 8.7% had a non-specified distribution of CMTC. Associated anomalies were observed in 42.5% of patients, predominantly body asymmetry and neurological defects like seizure and developmental delay. Fewer patients (10.1%) had ophthalmological defects, usually glaucoma. The major criterium “absence of venectasia” was not met in 20.4% of patients.

**Conclusion:**

We suggest that children with CMTC should be referred to an ophthalmologist for regular follow-up, and children with CMTC affecting the legs should be monitored for leg length discrepancy throughout the growth period. Furthermore, we suggest reconsideration of the major criterium “absence of venectasia” from the proposed diagnostic criteria, and instead include body asymmetry.

## Background

Cutis marmorata telangiectatica congenita (CMTC) is a rare congenital vascular anomaly, classified as a simple vascular malformation and subclassified as a capillary malformation (CM) by the International Society for the Study of Vascular Anomalies (ISSVA) [1]. CMTC is described as a persistent reticulated marbled erythema, which blanches with pressure and does not resolve with heating [2, 3]. As it affects capillaries and venules, CMTC is characterised as a slow-flow vascular lesion [4–6]. The affected cutaneous areas may develop cutaneous atrophy and ulcerations, and may also be associated with body asymmetry. The condition has often been reported as benign; however, associated anomalies such as congenital glaucoma, limb asymmetry and central nervous system involvement are frequently observed, which require the attention of medical professionals [7–10]. CMTC was first described by the Dutch paediatrician Cato van Lohuizen, who named the condition CMTC [11]. Since then, it has been referred to in the literature under several different terms including congenital generalised phlebectasia [12], naevus vascularis reticularis [13], congenital phlebectasia [14], congenital livedo reticularis [15] and van Lohuizen syndrome [16]. CMTC patients with co-existing Mongolian spots (“blue spots” or dermal melanocytosis) have been described as having phacomatosis pigmentovascularis type V (PPV type V) or phacomatosis cesiomarmorata [17–19].

Although the aetiology of CMTC remains unknown, two genetic theories were suggested by Rudolf Happle in 2002, who described the concept of an autosomal lethal mutation surviving by mosaicism and the theory of paradominant inheritance [20]. More recent studies identified *GNA11* mutations in skin biopsies from CMTC-affected skin areas [21–23]. In two of these studies, the mutation was either not detectable in blood [23] or found at a low level of 0.3% in blood [22]. CMTC is, however, still a clinical diagnosis [7–10]. Kienast et al. proposed a set of diagnostic criteria, where the presence of three major and two minor criteria out of five was considered indicative of CMTC [3]. The major criteria include: congenital reticulate (marmorated) erythema, absence of venectasia within the affected region at 1 year orof age, and unresponsiveness to local warming. Minor criteria are: fading of erythema within 2 years, telangiectasia within the CMTC-affected area, port-wine stain outside the CMTC-affected areas, ulceration, and cutaneous atrophy. However, these diagnostic criteria have not been validated. Histopathology does not play a role in the diagnosis of CMTC due to unspecific and inconsistent findings in skin biopsies [7, 24–26].

In this literature review, we evaluate the proposed criteria of Kienast et al. [3] and address the clinical manifestations, associated anomalies, differential diagnosis, management and prognosis of CMTC.

## Methods

**Fig. 1:**
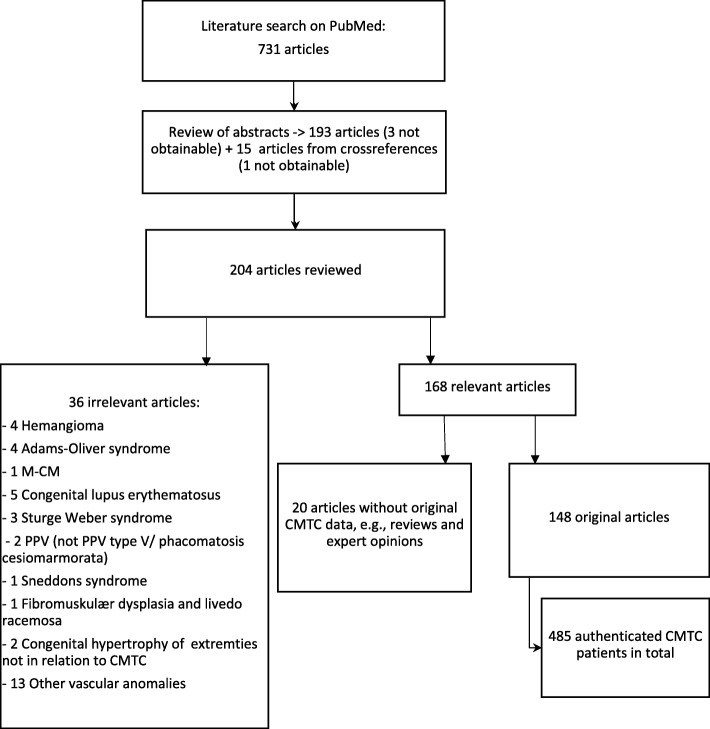
Flowchart illustrating the literature search for cutis marmorata telangiectatica congenita (CMTC) and the article selection process. The search was performed on April 17, 2019. M-CM, macrocephaly-capillary malformation. PPV, phacomatosis pigmentovascularis

A literature search was performed in PubMed using the following keywords: cutis marmorata telangiectatica congenita, Van Lohuizen’s syndrome, CMTC, congenital phlebectasia, naevus vascularis reticularis, congenital livedo reticularis, and phacomatosis cesiomarmorata. The MeSH search function in PubMed was also applied. The search retrieved 731 unfiltered articles. All abstracts for the unfiltered articles were reviewed in terms of their relevance to the subject, including synonyms for CMTC. We included articles written in English, German, French, Norwegian, Swedish, Danish and Turkish (Fig. 1).

A total of 193 articles were identified for full-text review. In addition, we searched the reference lists of the identified articles for additional sources, leading to a total of 204 articles for full-text review. A total of 168 articles were deemed relevant for the subject, including 148 original studies. Before exclusion of any articles, they were discussed among all authors.

In those articles with multiple cases consisting of both true CMTC patients and other capillary malformations such as macrocephaly-capillary malformation (M-CMTC, M-CM or M-CAP), Sturge Weber syndrome etc. only the true CMTC cases were included in the count. All uncertain cases were discussed in the study group, so only true CMTC cases were included in our study.

For each original article, the following variables were registered: gender, ethnicity, presence of the proposed diagnostic criteria of Kienast et al., distribution of skin lesions, associated anomalies, histopathology, family history, treatment, and prognosis.

## Results

### Patients

We identified 485 CMTC patients with skin lesions described from birth, within the first months of life or with an unspecified duration. Of these patients, 43.2% were male, 51.4% were female and 5.4% were an unspecified gender. The female:male ratio was 1.2:1. The patients represented different ethnicities including Caucasian, Hispanic, Asian, African and Middle Eastern. A total of seven CMTC cases were assumed to be familial [27–32].

Cutis marmorata was a prerequisite, but aside from this, the number of unavailable criteria according to Kienast et al. ranged from 66.0 to 88.2% (Table 1). Of the published CMTC patients, 20.4% had phlebectasia in affected skin areas. Among the minor criteria, the most frequent features were fading of erythema (29.5%), telangiectasia (16.7%), cutaneous atrophy (15.1%), port-wine stains (9.7%) and ulcerations (9.7%).

**Table 1:** Distribution of features according to the diagnostic criteria proposed by Kienast et al. [3]

	Patients positive for these features, n (%)	Patients negative for these features, n (%)	Information not available (N/A), n (%)
Major criteria			
• Congenital reticulate (marmorated) erythema	485 (100%)	0 (0%)	0 (0%)
• Absence of venectasia	4 (0.8%)	99 (20.4%)	382 (78.8%)
• Unresponsiveness to local heating	117 (24.1%)	0 (0%)	368 (75.9%)
Minor criteria			
•Fading of the erythema^a^	143 (29.5%)	22 (4.5%)	320 (66.0%)
•Telangiectasia	81 (16.7%)	8 (1.6%)	396 (81.6%)
•Port-wine stain	47 (9.7%)	10 (2.1%)	428 (88.2%)
•Ulcerations	47 (9.7%)	28 (5.8%)	410 (84.5%)
•Cutaneous atrophy	73 (15.1%0029	21 (4.3%)	391 (80.6%)

^a^Overall fading of the erythema, not just over a limited time period

We found that 24.5% of patients had generalised CMTC, 26.9% of whom had CMTC involving the face. A larger proportion of patients (66.8%) had localised CMTC, and 7.1% of these had CMTC erythema involving the face. Overall, the lower extremities were affected in 60.5% of patients, upper extremities in 25.9%, trunk in 27.5% and hands or feet in 4.9% (Table 2).

**Table 2:** Distribution of cutis marmorata telangiectatica congenita (CMTC)

	Number	Percentage
Generalised	119	24.5%
Generalised including face	32	26.9%
Localised	324	66.8%
Upper extremities	84	25.9%
Lower extremities	196	60.5%
Trunk	89	27.5%
Hand/foot	16	4.9%
Mucosa	2	0.6%
Face	23	7.1%
Not specified	42	8.7%
Total	485	

### Associated anomalies

A total of 206 patients (42.5%) had associated anomalies, 146 patients had no associated anomalies, and for the remaining 133 patients this information was not available. The most frequent anomaly was body asymmetry, seen in 37.7%. This includes asymmetry of the limbs, trunk and face as a result of either hypertrophy or hypotrophy. In addition, 10.1% had neurological defects, where the most frequent symptoms were seizures and developmental delay. The third most frequent anomaly was ophthalmological complications, seen in 9.9% of patients, half of which were congenital glaucoma. Furthermore, 5.2% had cardiovascular defects, 4.5% had Mongolian spots, 3.3% had dysmorphic features, 2.5% had genitourinary defects and 1.0% had endocrinological defects (Table 3).

**Table 3:** Distribution of associated abnormalities

	Patients positive for these features, n (%)	Patients negative for these features, n (%)	Information not available (N/A), n (%)
Body asymmetry Discrepancy in the girth and/or length of extremities, and hypo/hypertrophy of trunk and face.	183 (37.7%)	38 (7.8%)	264 (54.4%)
Neurological defects Developmental delay, seizures, epilepsy, brachy plagiocephaly, cerebral atrophy, arteriovenous malformation of the brain, mental retardation, transient ischemic attack, triventricular hydrocephalus, corpus callosum agenesis, white matter calcification, hemiparesis, hemispheric vascular anomaly, hearing impairment, dyscrania, microcephalia, and porencephaly.	49 (10.1%)	57 (11.8%)	380 (78.4%)
Ophthalmological defects Glaucoma, blue pigmentation on the sclera, cornea and conjunctiva, retinal vascular abnormalities, retinal detachment, amblyopia, and retinoblastoma.	48 (9.9%)	53 (10.9%)	385 (79.4%)
Cardiovascular defects Cardiac malformation, predominantly atrial-septal defect and patent ductus arteriosus, hypertension, and sinus arrhythmia.	25 (5.2%)	31 (6.4%)	430 (88.7%)
Mongolian spots Blue spots.	22 (4.5%)	2 (0.4%)	462 (95.3%)
Dysmorphic features Syndactyly, micrognathia, widely spread toes, hypertelorism, frontal bossing, flat face, low-set ears, club foot, cleft palate, and epicanthal folds.	16 (3.3%)	6 (1.2%)	464 (95.7%)
Genitourinary defects Hypospadias, double ureter, undescended testis, hydrocele, cryptorchidism, urethral obstruction, and clitoral/urethral meatus agenesis.	12 (2.5%)	1 (0.2%)	473 (97.5%)
Abdominal defects Hepatosplenomegaly, imperforate anus, neonatal ascites, gastro-oesophageal reflux, and malrotated bowel.	11 (2.3%)	22 (4.5%)	453 (93.4%)
Nephrological defects Hydronephrosis, renal hypoplasia, and multi-cystic renal disease.	10 (2.1%)	11 (2.3%)	465 (95.9%)
Endocrinological defects Hypothyroidism, hyperlipidaemia, and abnormal copper metabolism.	5 (1.0%)	5 (1.0%)	476 (98.1%)

Most frequent conditions under each category are listed first

### Glaucoma

**Fig. 2:**
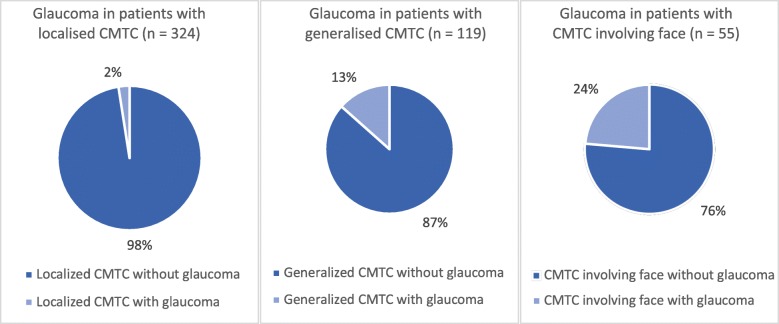
Distribution of glaucoma in patients with cutis marmorata telangiectatica congenita (CMTC)

Twenty-four (4.9%) of all 485 published CMTC patients had glaucoma. Patients with generalised CMTC had a higher tendency for glaucoma, which was present in 16 (13.4%) out of 119 patients with generalised CMTC. Of the patients with localised CMTC, eight patients (2.5%) had glaucoma out of 324 patients with localised CMTC. Patients with CMTC on the face had the highest frequency of glaucoma, comprising 13 (24%) out of 55 patients with CMTC on the face (Fig. 2).

### Leg length discrepancy

**Fig. 3:**
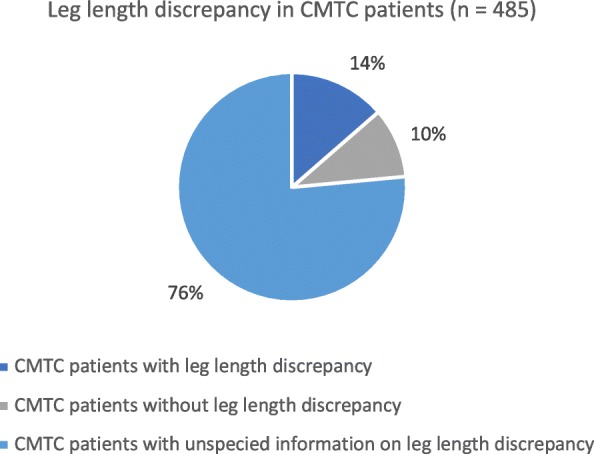
Distribution of leg length discrepancy in patients with cutis marmorata telangiectatica congenita (CMTC)

Body asymmetry was observed in 37.7% of CMTC patients, and of those, 36.1% had a leg length discrepancy. Of all of the 485 CMTC patients, 13.6% (Fig. 3) had a leg length discrepancy ranging from 1 to 6.8 cm.

## Discussion

We applied the proposed diagnostic criteria of Kienast et al. [3] to assess CMTC. One of the major criteria is the absence of venectasia; however, we found that phlebectasia was present in 20.4% of the published CMTC patients, suggesting that this major criteria should be reconsidered. Evaluating the diagnostic criteria of Kienast et al. in terms of validity is a challenge due to both the retrospective nature of this review and the amount of missing information for the published patients, ranging from 66.0–88.2%.

Improvement in the marbled skin appearance over time was described in 143 (29.5%) patients (Table 1), whereas 4.5% did not show improvement, and this information was lacking in the remaining 66.0% of patients. There was a wide age span for when improvement was seen. The literature reported that patients between the ages of 6 weeks to 26 years showed improvement of the skin condition [2, 33]; however, many articles did not specify the exact age at improvement. In several articles, improvement was described after 2 years of age [7, 34–37]. Patients with no improvement of CMTC were also described [15, 17, 38–43], but some of these patients had a short follow-up [44–46]. Adults with persistent erythema were also reported [27, 47, 48]. Another issue in the literature was the short follow-up or no follow-up, which makes it challenging to describe the precise prognosis of CMTC.

In the literature, asymmetry was found to be the most frequent anomaly, comprising 37.7%, while the minor criteria “improvement of erythema” should be further investigated as a part of CMTC in future prospective studies.

### Associated anomalies

The definition of associated anomalies varies in the literature. Some authors regard cutaneous atrophy, ulcerations and port-wine stains as associated anomalies rather than integral parts of the syndrome, therefore the percentage of CMCT patients reported to have associated anomalies ranges from 18.8 to 80% [3, 7, 8, 10, 24, 29, 49]. In this study, we defined associated anomalies as those not included in the proposed diagnostic criteria (Table 3), and found that 42.5% of the CMTC patients had associated anomalies. However, this finding might be an overestimation due to publication bias, and it might not reflect the true nature of CMTC. We recognise that many findings of associated anomalies listed in Table 3 may be coincidental findings.

### Glaucoma

There was a relatively high presence of glaucoma, reported in 4.9% of patients. In patients with generalised CMTC, the proportion increased to 13.4%, and in patients with skin lesions on the face, the proportion was 24%. Even though glaucoma is not the most frequent anomaly, it can have severe consequences including decreased vision and, in the worst case, blindness, which may occur if it is not discovered in time. Most CMTC patients were diagnosed with glaucoma in early infancy [50–54]; however, two patients were described to have late-onset glaucoma at the age of 3 and 9 years despite earlier ophthalmological check-ups [55, 56]. The nature of this rare condition and the consequences of overlooked glaucoma suggest that CMTC patients should be referred to and followed up by an ophthalmologist.

### Leg length discrepancy

Body asymmetry was the most frequent associated anomaly, and 13.6% of all reported patients had a leg length discrepancy. This defect can have functional consequences if not treated timely. One patient was described to have a leg length discrepancy that resolved spontaneously within the first 9 months of life [46]. Another patient, however, had a leg length discrepancy which progressed over time at 6 and 9 months of follow-up [57]. In another patient, growth retardation of one leg was first noticed at 6 months of age [58]. This suggests that children with CMTC affecting the lower extremities should be monitored for leg length discrepancy during childhood.

A large study from 2014 including a total of 29 patients with CMTC and leg length discrepancy suggested a treatment algorithm where leg length discrepancy greater than 2 cm should be treated with epiphysiodesis [9].

### Differential diagnosis

The characteristic marbled erythema of CMTC can also be seen in other conditions such as those listed in Table 4. Some of these differential diagnoses have a more severe prognosis and require different treatment approaches, such as Klippel-Trenaunay syndrome and macrocephaly-capillary malformation, which highlights the importance of a correct diagnosis.

**Table 4:** Differential diagnoses and distinguishing clinical features

Condition	Distinguishing clinical features
Physiological cutis marmorata	Symmetric blanchable and reticulate pattern on the trunk and extremities which disappear with local warming.
Congenital livedo reticularis	Idiopathic or secondary to Down’s syndrome, Cornelia de Lange syndrome, neonatal lupus erythematosus, antiphospholipid antibody syndrome, vasculopathies or autoimmune connective tissue disorders.
Klippel-Trenaunay syndrome [1]	Soft tissue and bone hypertrophy with port-wine stain, lymphangioma, and/or varicosities typically involving one extremity. Associated with *PIK3CA* mutation.
Sturge-Weber syndrome [1]	Facial port-wine stain, vascular malformation in eyes and meninges, and calcium deposits in the brain. Many of the patients have mutations in the *GNAQ* gene.
Macrocephaly-capillary malformation (formerly macrocephaly-cutis marmorata telangiectatica congenita) [1, 59]	Macrocephaly often with developmental delay. Somatic mutation in the *PIK3CA* gene.
Sneddon’s syndrome [60]	Cerebrovascular ischemic events and generalised livedo racemosa. Histopathology shows occlusive arteriopathy and endothelial damage.
Parkes-Weber syndrome [1, 6]	Extremity hypertrophy containing arterial-venous fistula and hemangiomas. Associated with *RASA1* mutations.
Adams-Oliver syndrome [61]	Cardiac malformations, limb defects, aplasia cutis congenita of the scalp and abnormalities of the cranium.
Genuine diffuse phlebectasia (Bockenheimer’s disease) [62]	Progressive congenital phlebectasia, usually on a single extremity.

### Genetics

The recent genetic finding of *GNA11* mutation in affected skin [21–23] confirms that CMTC is possibly a postzygotic mosaic condition. This explains the low incidence of familial cases. Two other studies have reported autosomal recessive inherited homozygous mutations in the *ARL6IP6* gene in patients with CMTC and stroke [38, 63]. The consequence of the *ARL6IP6* gene mutation remains unknown, although it is thought to be a genetic susceptibility factor for younger patients with ischemic stroke [64].

### Treatment and follow-up

When suspicion of CMTC is raised, it is recommended to perform a careful evaluation of the patient for associated anomalies, ideally in a multidisciplinary team with a paediatrician, dermatologist, ophthalmologist and, eventually, an orthopaedic surgeon.

Two reports described effective laser therapy for erythema and ulceration [23, 65], while other reports stated no effect of laser treatment [25, 66]. A single case of ineffective left brachial sympathectomy can also be found in the literature [27]. Finally, another patient received sympathetic nerve blockade combined with vasodilator treatment with good efficacy for pain in CMTC-affected areas [67]. Due to the limited number of studies, we cannot recommend a treatment strategy for skin lesions in CMTC.

## Conclusion

**Fig. 4:**
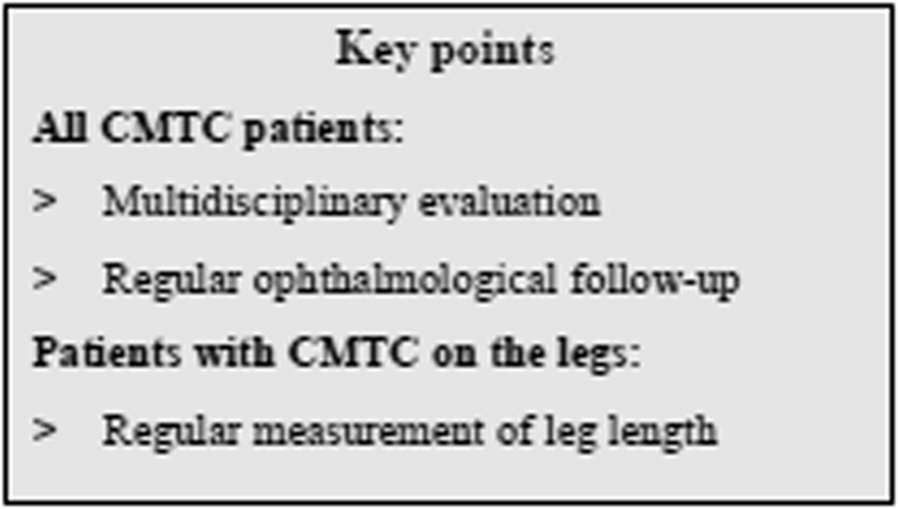
Management of patients with cutis marmorata telangiectatica congenita (CMTC)

CMTC is a relatively benign disorder on its own, which does not usually require treatment. However, health care professionals should be aware of the frequently associated anomalies, such as glaucoma and leg length discrepancy, which may have serious consequences if not recognised and treated. We suggest that children with CMTC should be referred to an ophthalmologist after birth for regular check-ups for glaucoma, and that children with CMCT on the legs should be regularly monitored for leg length discrepancy during childhood until the end of the growth period (Fig. 4). Furthermore, we suggest reconsideration of the major criteria “absence of venectasia” from the proposed diagnostic criteria, and propose that body asymmetry should be taken into consideration. Finally, the genetic research in this area is evolving, most recently with the identification of mutations in the *GNA11* gene. Further studies should clarify whether molecular genetics should be part of the diagnostic process in the future.

## Data Availability

All data used for this paper was from publicly available sources (PubMed).

## Abbreviations

CMTC: Cutis marmorata telangiectatica congenita
M-CM: Macrocephaly-Capillary Malformation
PPV: Phacomatosis Pigmentovascularis

## References

[1] International Society for the Study of Vascular Anomalies (2018). ISSVA Classification of Vascular Anomalies.

[2] Pehr K, Moroz B (1993). Cutis marmorata telangiectatica congenita: long-term follow-up, review of the literature, and report of a case in conjunction with congenital hypothyroidism. Pediatr Dermatol.

[3] Kienast AK, Hoeger PH (2009). Cutis marmorata telangiectatica congenita: a prospective study of 27 cases and review of the literature with proposal of diagnostic criteria. Clin Exp Dermatol.

[4] Bormann G, Wohlrab J, Fischer M, Marsch WC (2001). Cutis marmorata telangiectatica congenita: laser doppler fluxmetry evidence for a functional nervous defect. Pediatr Dermatol.

[5] Perman MJ, Castelo-Soccio L, Jen M (2012). Differential diagnosis of infantile hemangiomas. Pediatr Ann.

[6] Redondo P, Aguado L, Martinez-Cuesta A (2011). Diagnosis and management of extensive vascular malformations of the lower limb: part I. Clinical diagnosis J Am Acad Dermatol.

[7] Amitai DB, Fichman S, Merlob P, Morad Y, Lapidoth M, Metzker A (2000). Cutis marmorata telangiectatica congenita: clinical findings in 85 patients. Pediatr Dermatol.

[8] Devillers AC, de Waard-van der Spek FB, Oranje AP (1999). Cutis marmorata telangiectatica congenita: clinical features in 35 cases. Arch Dermatol.

[9] Memarzadeh A, Pengas I, Syed S, Eastwood DM (2014). Limb length discrepancy in cutis marmorata telangiectatica congenita: an audit of assessment and management in a multidisciplinary setting. Br J Dermatol.

[10] Gerritsen MJ, Steijlen PM, Brunner HG, Rieu P (2000). Cutis marmorata telangiectatica congenita: report of 18 cases. Br J Dermatol.

[11] Van Lohuizen C (1922). Über eine seltene angeborene Hautanomalie (Cutis marmorata telangiectatica congenita). Acta Dermatol Venereol.

[12] Kantor I, Yep D (1960). Congenital generalized phlebectasia. Arch Dermatol.

[13] Brain RT (1954). Naevus vascularis reticularis: two cases. Proc R Soc Med.

[14] Lynch PJ, Zelickson AS (1967). Congenital phlebectasia. A histopathologic study. Arch Dermatol.

[15] Champion RH (1965). Livedo reticularis. A review Br J Dermatol.

[16] Lee S, Lee JB, Kim JH, Kim KY, Lee SH (1981). Cutis marmorata telangiectatica with multiple congenital anomalies (van Lohuizen’s syndrome). Dermatologica.

[17] Torrelo A, Zambrano A, Happle R (2003). Cutis marmorata telangiectatica congenita and extensive mongolian spots: type 5 phacomatosis pigmentovascularis. Br J Dermatol.

[18] Torrelo A, Zambrano A, Happle R (2006). Large aberrant Mongolian spots coexisting with cutis marmorata telangiectatica congenita (phacomatosis pigmentovascularis type V or phacomatosis cesiomarmorata). J Eur Acad Dermatol Venereol.

[19] Happle R (2005). Phacomatosis pigmentovascularis revisited and reclassified. Arch Dermatol.

[20] Dohi HR, Lecture M (2002). New aspects of cutaneous mosaicism. J Dermatol.

[21] Sassalos TM, Fields TS, Levine R, Gao H. Retinal neovascularization from a patient with cutis marmorata telangiectatica congenita. Retin Cases Brief Rep. 2018. 10.1097/ICB.0000000000000736. 10.1097/ICB.0000000000000736 29543621

[22] Thomas FP, Guergueltcheva V, Gondim FA, Tournev I, Rao CV, Ishpekova B (2016). Clinical, neurophysiological and morphological study of dominant intermediate Charcot-Marie-tooth type C neuropathy. J Neurol.

[23] Kumar A, Zastrow DB, Kravets EJ, Beleford D, Ruzhnikov MRZ, Grove ME, et al. Extracutaneous manifestations in phacomatosis cesioflammea and cesiomarmorata: case series and literature review. Am J Med Genet A. 2019;179(6):966–77. 10.1002/ajmg.a.61134 PMC6488410 30920161

[24] South DA, Jacobs AH (1978). Cutis marmorata telangiectatica congenita (congenital generalized phlebectasia). J Pediatr.

[25] Mazereeuw-Hautier J, Carel-Caneppele S, Bonafe JL (2002). Cutis marmorata telangiectatica congenita: report of two persistent cases. Pediatr Dermatol.

[26] Hinek A, Jain S, Taylor G, Nykanen D, Chitayat D (2008). High copper levels and increased elastolysis in a patient with cutis marmorata teleangiectasia congenita. Am J Med Genet A.

[27] Andreev VC, Pramatarov K (1979). Cutis mamorata telangiectatica congenita in two sisters. Br J Dermatol.

[28] Bjornsdottir US, Laxdal T, Bjornsson J (1988). Cutis marmorata telangiectatica congenita with terminal transverse limb defects. Acta Paediatr Scand.

[29] Picascia DD, Esterly NB (1989). Cutis marmorata telangiectatica congenita: report of 22 cases. J Am Acad Dermatol.

[30] Kurczynski TW (1982). Hereditary cutis marmorata telangiectatica congenita. Pediatrics.

[31] Lunge SB, Mahajan P (2014). Cutis marmorata telangiectatica congenita restricted to both breasts in a young female. Dermatol Pract Concept.

[32] Taleb EA, Nagpal MP, Mehrotra NS, Bhatt K (2018). Retinal findings in a case of presumed cutis marmorata telangiectatica congenita. Retin Cases Brief Rep..

[33] Nicholls DS, Harper JI (1989). Cutis marmorata telangiectatica congenita with soft-tissue herniations on the lower legs. Clin Exp Dermatol.

[34] Altman AR, Tschen JA, Wolf JE (1984). Cutis marmorata telangiectatica congenita: a case report. Pediatr Dermatol.

[35] Humphries JM (1952). Generalized congenital phlebectasia. J Pediatr.

[36] Nyrnes SA, Vesterhus P, Johnsen PO (2003). Cutis marmorata telangiectatica congenita. Tidsskr Nor Laegeforen.

[37] Spitzer MS, Szurman P, Rohrbach JM, Aisenbrey S (2007). Bilateral congenital glaucoma in a child with cutis marmorata telangiectatica congenita: a case report. Klin Monatsbl Augenheilkd.

[38] Abumansour IS, Hijazi H, Alazmi A, Alzahrani F, Bashiri FA, Hassan H (2015). ARL6IP6, a susceptibility locus for ischemic stroke, is mutated in a patient with syndromic cutis marmorata telangiectatica congenita. Hum Genet.

[39] Avci S, Calikoglu E, Sayli U (2001). Cutis marmorata telangiectatica congenita: an unusual cause of lower extremity hypoplasia. Turk J Pediatr.

[40] Balazsi G, Polomeno RC, Duperrem J (1990). New findings related to IOP elevation in CMTC. J Pediatr Ophthalmol Strabismus.

[41] Carrascosa JM, Ribera M, Bielsa I, Coroleu W, Ferrandiz C (1996). Cutis marmorata telangiectatica congenita or neonatal lupus?. Pediatr Dermatol.

[42] Chang BP, Hsu CH, Chen HC, Hsieh JW (2007). An infant with extensive Mongolian spot, naevus flammeus and cutis marmorata telangiectatica congenita: a unique case of phakomatosis pigmentovascularis. Br J Dermatol.

[43] Mizrahi AM, Sachs PM (1966). Generalized congenital phlebectasia. Report of a case. Am J Dis Child.

[44] Fujita M, Darmstadt GL, Dinulos JG (2003). Cutis marmorata telangiectatica congenita with hemangiomatous histopathologic features. J Am Acad Dermatol.

[45] Schultz RB, Kocoshis S (1979). Cutis marmorata telangiectatica congenita and neonatal ascites. J Pediatr.

[46] Byrom L, Surjana D, Yoong C, Zappala T. Red-white and blue baby: a case of phacomatosis pigmentovascularis type V. Dermatol Online J. 2015;21(6). 26158369

[47] Baxter P, Gardner-Medwin D, Green SH, Moss C (1993). Congenital livedo reticularis and recurrent stroke-like episodes. Dev Med Child Neurol.

[48] Miller CL, Fuseler JW, Brinkley BR (1977). Cytoplasmic microtubules in transformed mouse x nontransformed human cell hybrids: correlation with in vitro growth. Cell.

[49] Kennedy C, Oranje AP, Keizer K, van den Heuvel MM, Catsman-Berrevoets CE (1992). Cutis marmorata telangiectatica congenita. Int J Dermatol.

[50] Miranda I, Alonso MJ, Jimenez M, Tomas-Barberan S, Ferro M, Ruiz R (1990). Cutis marmorata telangiectatica congenita and glaucoma. Ophthalmic Paediatr Genet.

[51] Mayatepek E, Krastel H, Volcker HE, Pfau B, Almasan K (1991). Congenital glaucoma in cutis marmorata teleangiectatica congenita. Ophthalmologica.

[52] Sato SE, Herschler J, Lynch PJ, Hodes BL, Fryczkowski AW, Schlosser HD (1988). Congenital glaucoma associated with cutis marmorata telangiectatica congenita: two case reports. J Pediatr Ophthalmol Strabismus.

[53] Cremer H (1982). Cutis marmorata telangiectatica congenita. Hautarzt.

[54] Lynch PJ (1990). Cutis marmorata telangiectatica congenita associated with congenital glaucoma. J Am Acad Dermatol.

[55] Pendergast SD, Trese MT, Shastry BS (1997). Ocular findings in cutis marmorata telangiectatica congenita. Bilateral exudative vitreoretinopathy. Retina.

[56] Murphy CC, Khong CH, Ward WJ, Morgan WH (2007). Late-onset pediatric glaucoma associated with cutis marmorata telangiectatica congenita managed with Molteno implant surgery: case report and review of the literature. J AAPOS.

[57] Amaral J, Peixoto S, Mimoso G, Pereira D. Cutis marmorata telangiectatica congenita and major lower limb asymmetry. BMJ Case Rep. 2018;2018. 10.1136/bcr-2017-222269. 10.1136/bcr-2017-222269 PMC5780593 29330270

[58] Imafuku S, Tashiro A, Furue M, Nakayama J (2008). Cutis marmorata telangiectatica congenita manifesting as port-wine stain at birth. J Dermatol.

[59] Wright DR, Frieden IJ, Orlow SJ, Shin HT, Chamlin S, Schaffer JV (2009). The misnomer “macrocephaly-cutis marmorata telangiectatica congenita syndrome”: report of 12 new cases and support for revising the name to macrocephaly-capillary malformations. Arch Dermatol.

[60] Wohlrab J, Fischer M, Wolter M, Marsch WC (2001). Diagnostic impact and sensitivity of skin biopsies in Sneddon’s syndrome. A report of 15 cases. Br J Dermatol.

[61] Baskar S, Kulkarni ML, Kulkarni AM, Vittalrao S, Kulkarni PM (2009). Adams-Oliver syndrome: Additions to the clinical features and possible role of BMP pathway. Am J Med Genet A.

[62] van Geest AJ, Veraart JC, de Haan M, Neumann HA (1999). Bockenheimer’s syndrome. J Eur Acad Dermatol Venereol.

[63] Dereure O (2016). Cutis marmorata telangiectatica congenita: mutations in a susceptibility gene involved in cerebrovascular accidents. Ann Dermatol Venereol.

[64] Cheng YC, O’Connell JR, Cole JW, Stine OC, Dueker N, McArdle PF (2011). Genome-wide association analysis of ischemic stroke in young adults. G3 (Bethesda).

[65] Deshpande AJ (2018). Cutis mormorata telangiectatica congenital successfully treated with intense pulsed light therapy: a case report. J Cosmet Laser Ther.

[66] Adachi K, Togashi S, Sasaki K, Sekido M (2013). Laser therapy treatment of phacomatosis pigmentovascularis type II: two case reports. J Med Case Rep.

[67] Sahin A, Celebi N, Dogan R, Canbay O, Uzumcugil F, Aypar U (2006). Lumbar sympathetic blockade in a patient with cutis marmorata telangiectatica congenita. Paediatr Anaesth.

